# Comparison of Dimensional Accuracy between Open-Tray and Closed-Tray Implant Impression Technique in 15° Angled Implants

**Published:** 2013-09

**Authors:** F Balouch, E Jalalian, M Nikkheslat, R Ghavamian, Sh Toopchi, F Jallalian, S Jalalian

**Affiliations:** a Dept. of Prosthodontics, Dental Branch, Islamic Azad University, Tehran, Iran.; b DDS; c Dental Student, Dental Branch, Islamic Azad University. Tehran, Iran.; d Post Graduate Student, Dept. of Radiology, Dental Faculty, Isfahan University of Medical Sciences, Isfahan, Iran.; e Post Graduate Student, Dept. of Prosthodontics, Dental Branch, Islamic Azad University, Tehran, Iran.

**Keywords:** Implant, Impression, Open Tray, Closed Tray, Dimensional Changes

## Abstract

**Statement of Problem: **Various impression techniques have different effects on the accuracy of final cast dimensions. Meanwhile; there are some controversies about the best technique.

**Purpose: **This study was performed to compare two kinds of implant impression methods (open tray and closed tray) on 15 degree angled implants.

**Materials and Method:** In this experimental study, a steel model with 8 cm in diameter and 3 cm in height were produced with 3 holes devised inside to stabilize 3 implants. The central implant was straight and the other two implants were 15° angled. The two angled implants had 5 cm distance from each other and 3.5 cm from the central implant. Dental stone, high strength (type IV) was used for the main casts. Impression trays were filled with poly ether, and then the two impression techniques (open tray and closed tray) were compared. To evaluate positions of the implants, each cast was analyzed by CMM device in 3 dimensions (x,y,z). Differences in the measurements obtained from final casts and laboratory model were analyzed using t-Test.

**Results: **The obtained results indicated that closed tray impression technique was significantly different in dimensional accuracy when compared with open tray method. Dimensional changes were 129 ± 37μ and 143.5 ± 43.67μ in closed tray and open tray, while coefficient of variation in closed- tray and open tray were reported to be 27.2% and 30.4%, respectively.

**Conclusion: **Closed impression technique had less dimensional changes in comparison with open tray method, so this study suggests that closed tray impression technique is more accurate.

## Introduction

Dimensional changes occur due to the contraction in the impression material which is initiated by polymerization reaction with formation of volatile materials and by-products, pressure applied during impression and conventional impression techniques. Making a precise mold of implant is necessary for passive fitness. Passive fitness is the term used to address fitting status of the implant in which implant body shows adequate fitting for simultaneous adaptation and remodeling [1].

Making a superstructure with passive fitness is one of the main objectives during implant-based prosthesis. Preparation of a precise mold with stable dimensions prior to casting is necessary to achieve this passive fitness [2]. However, failing to achieve this passive fitness will incur stress on implants which can finally lead to fracture of the implant components and failure of the treatment. The forces created in the implant due to non-passive nature of the superstructure is able to resorb the bone surrounding the implant and cause ischemia within peri-implant tissue and subsequent healing with non-mineral tissue around the implant, mechanical fracture, loosening of the implant components and fracture of the restoration [3].

There are several methods to achieve passive fitness, although no distinct protocol has been introduced in this field yet [4]. It is now believed that the impression materials are significantly improved, so choosing the proper technique would be the main issue [3].

Recent developments in impression techniques, to obtain the maximum accuracy of the implant position, have been regarded more than other issues [5]. Some degrees of error and inaccuracy have also been noticed in the precise transfer of the implant positions for all impression methods. The most common techniques are closed- tray, open- tray which have been cited almost similarly in the literature, although angulation of the implants plays a key role in the accuracy of impression [5]. The transfer technique uses tapered copings and a closed tray to make an impression.

The copings are connected to the implants, and an impression is made and removed from the mouth, leaving the copings in the mouth. Subsequently, the copings are removed and connected to the implant analogs, and then the coping-analog assemblies are inserted in the impression before pouring the definitive cast. The clinical situations which indicate the use of the closed tray technique are when the patient has limited interarch space, tendency to gag, or if it is too difficult to access an implant in the posterior region of the mouth and angulated implants [6]. 

Conversely, the pick-up impression uses square copings and an open tray (a tray with an opening), allowing the coronal ends of the impression coping screw to be exposed. Before separating the implants, the copings screws are unscrewed to be removed along with the impression.

The implant analogs in the impression are connected to the copings to fabricate the definitive cast. The disadvantages of this technique could be that there may be some rotational movement of the impression coping when securing the implant analog, and blind attachment of the implant analog to the impression coping may result in a misfit of components [8]. 

It has been claimed by other research that the closed tray technique is more accurate for impression single implant [9]. Moreover, one other study has claimed that the closed tray technique is preferred even for three implants [10]. Indirect methods are often clinically preferred by clinicians [2, 11].

With considering mentioned contradictions, this research aims to compare two impression methods of open tray and closed tray in dimensional accuracy for 15° angle implants. 

## Materials and Method

**Figure 1:**
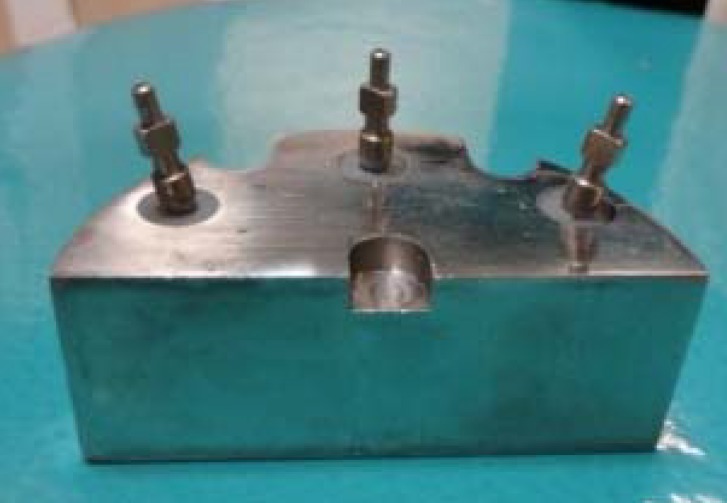
Master model (the control group)

The research was done through an experimental-laboratory method on 15 input samples in each group, forming a total number of 31 samples. In which each sample has 6 points (A, B, C, D, E, F). A steel model, having 8 cm diameter and 3 cm height, was produced first and then three grooves were devised for three implants (Noble Biocare; AB, Göteborg Sweden). Every two angulated implants were 5 cm apart with 3.5 cm distance from central implant. The position of implants was analyzed by the surveyor so the central implant was placed perpendicular to the casting surface while the other implants had divergence or convergence of 15° from the central component (Figure 1). 

**Figure 2:**
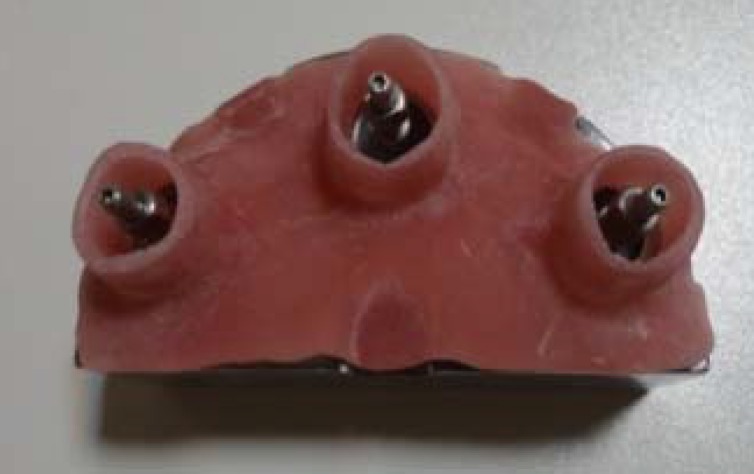
Acrylic customized open tray

For making the main cast, dental stone, high strength (type IV, Ernest hinritchs; Germany) was used in a vacuumed mixer apparatus. The whole implant was fixed using cyanoacrylate. All operations were implemented by one operator. Trays were prepared from polymerizable acryl in visible light (megadenta; Germany) and polymerized for 6 minutes (Figure 2). The trays were then trimmed and perforated to enhance gripping of the impression material. Meanwhile, the main cast was equipped with two guide pins in order to fit the designed tray using open tray method.

**Figure 3:**
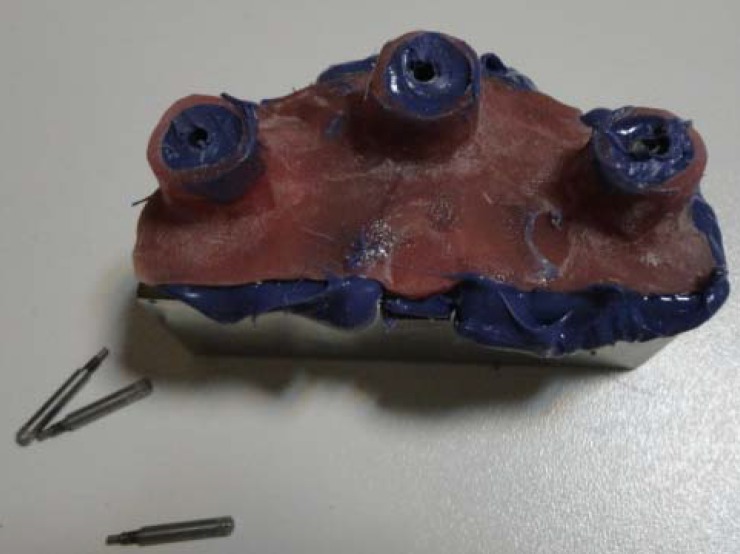
Open tray impression

The trays were filled by polyether (IMPREGUM sm-espe; Germany), placed on the main cast and the additional material was removed by finger from the perforations to uncover pins. The impression material was allowed to be polymerized for 10 minutes before detachment (Figure 3). 

In open tray technique, the guide pins were loosened using hex-driver and were removed. Then, the tray was detached from the main cast with the copings being remained in the mold, while analog of the implant was connected to the impression copings. Impression copings of the closed tray remained on the main cast after polymerization of the impression material. These copings were removed from the main cast and connected to the analog when the tray was removed. Analog units of the compound coping were placed deep in the impression by applying pressure with complete or partial clockwise rotation till a resistance against rotation was felt. This contact feeling implies that position of the implant has been correctly transferred.

**Figure 4:**
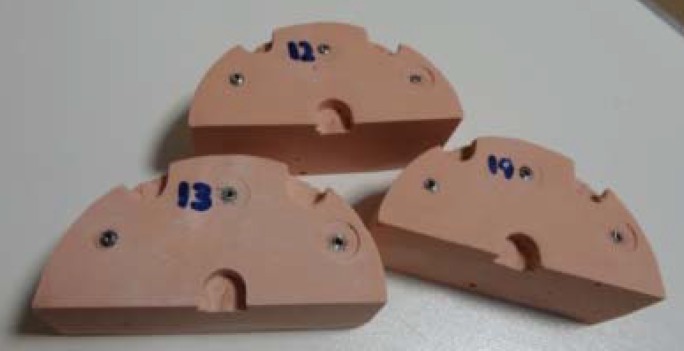
Dental stone, high strength (type IV) casts (experimental group)

The impression was examined and it was repeated when any kind of deficiency was observed including trapped air bubbles and leftovers of impression material between the coping connection and the analog. Dental stone, high strength (type IV) cast was then prepared according to instructions of the manufacturer. Casts were trimmed and coded after being cured for one hour (Figure 4). 

**Figure 5:**
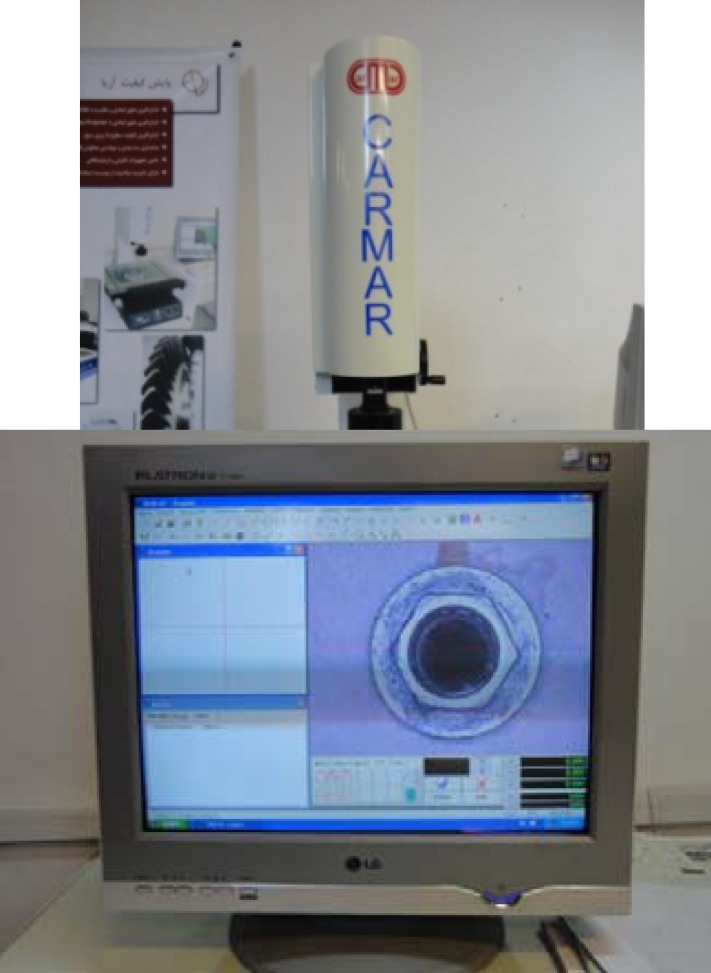
CMM Devices for evaluating dimensional changes

The casts were analyzed by CMM (coordinating measuring machine) devices (DEA; Italy) in three dimensions (x, y, and z) for evaluating the position of implants and dimensional changes. Therefore, they were fixed on a mounting plate using a vise for further measurements. A fine tip stylus was then adopted to record multi-axial coordinators (x, y, z) on the upper surface of hex implant and also on the casting base. The tip of the stylus was located on the center of the hex implants and calculated for six vertices of the hex and three plans (x, y, and z). Afterwards, different vector calculations were determined, in degrees, between implant angles on both the main and the duplicate cast [11-13] (Figure 5). Statistical analysis adopted in this study was Student t-test.

## Results

The current study was performed on a total of 31 samples which had 15 test samples in each group in addition to six defined points (A, B, C, D, E, F). The content of dimensional changes in transfer of implant positions was reported to be 129±37μm for experimental groups (closed tray) and 143.5±43.6μm for the (open tray) (Table 1). 

**Figure 6:**
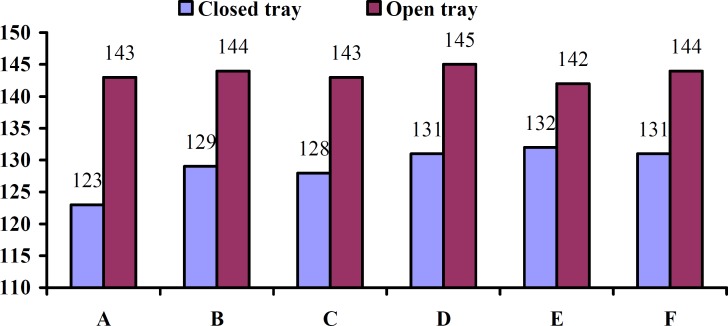
Dimensional changes (µ) in 6 points (A, B, C, D, E, F).

**Table 1:** Mean dimensional changes (µ) in open- tray and closed- tray methods

**Methods**	**Open Tray**	**Closed Tray**	**P.value**
**Groups**			
A (n=31)	143 ± 41.7	123 ± 35.1	*p*< 0.02
B (n=31)	144 ± 42.6	129 ± 35.5	*p*< 0.07
C (n=31)	143 ± 44.4	128 ± 38.1	*p*< 0.09
D (n=31)	145 ± 46	131 ± 36.6	*p*< 0.2
E (n=31)	142 ± 44.6	132 ± 38.9	*p*< 0.2
F (n=31)	144 ± 42.7	131 ± 38.2	*p*< 0.2

Thus, the values of the latter group were 14.5μm or 11.2% greater than the former one. Results of Student’s t-test indicated that the changes for open tray group were significantly greater than the close tray (*p*< 0.001). Meanwhile, coefficients of the variations were calculated to be 28.7% and 30.4% for the experimental groups, respectively. 

Dimensional changes in the transfer of implant positions have been displayed in figure 6 based on the points under study and the impression methods applied. They show that changes in open tray method were greater than closed tray (in all points) and it was statistically significant (*p*< 0.09). 

## Discussion

The obtained results indicated that closed tray impression method had lesser dimensional changes and it was more acceptable than open tray technique.

The current study revealed that the dimensional changes for closed tray were lesser than the open tray impression method in the implants with 15° divergence position. This finding contradicts with the results of the most previous researches and the cited references.

Some studies in literature have not distinguished the differences as significant [1, 10-11], whereas some others have identified open tray impression method more accurate [2]. However, dimensional changes of closed tray technique which was lesser in this study can be attributed to its simplicity, accuracy of operator in implementing the technique [2, 14] and application of custom tray instead of prefabricated tray [5]. Regarding the inconsistent results on preference of either direct (open tray) or indirect (closed tray) method, it seems that the accuracy of the operator has been more effective than any other factor in final results.

Rismanchian and Moniri Fard [2] applied two methods of programmed search and manual search in several relevant journals to study articles associated with implant impression, common methods and modifications made. They finally concluded that direct impression (open tray) method was more accurate than indirect (closed tray) method.

 Most researchers have preferred the direct methods and have confined indirect techniques to single implant applications. Although, some others have clinically verified both techniques in spite of their different statistical significance and have identified the errors as tolerable [15].

Seyyedan et al. [1] had noticed passive fitness as one of the prerequisites of implant supported prosthesis. They did not distinguish a significant difference between open tray and closed tray techniques. Also angulations of the implants were not mentioned in their research [1].

Based on the study enrolled by Carbal and coworkers which analyzed four different impression techniques for implants; different methods were investigated and compared concerning their dimensional accuracy [15]. The results were compared with standard technique of this research and did not reveal a significant relationship between the two impression methods of direct and indirect. Their research also failed to consider angulations of the implants.

Based on a research enrolled by Heather et al.; accuracies of two impression techniques, namely open tray and closed tray, were not significantly different (*p*= 0.22) [16]. They also noticed that implant angle and implant number were different from average error of angulations but not large enough to be simply interpreted (*p*> 0.001). Various angles of 5°, 10° and 15° were experienced in their research although the measurement time after casting and the software used werenot verified.

The study of Daudi et al. investigated the accuracy of the two impression techniques for single implants in laboratory [17] and focused on the accuracy of four impression processes of implants through direct and indirect methods; using poly ether and poly vinyl siloxane materials. The SAS software was utilized in their work which identified the indirect method as more preferable.

Walker et al. worked on the accuracy of implant casting as a function of impression technique and viscosity of the impression material [18]. They demonstrated that casts built through closed tray (indirect) method with metallic copings on the surface of the implant were more accurate than the casts fabricated by open tray (direct) method with plastic copings.

Current study has benefited from the best 3-D measurement equipments with the highest possible resolution in small dimensions. One instance is using the coordinating measurement tools such as CMM [1, 5].

A significant difference was observed in this research between straight and angulated implants. Angulated implants incur a great stress to the impression materials once the molds are being removed out of mouth which can cause permanent deformations in these materials.

Most research has focused on the accuracy of techniques with parallel implants [19-31] but nonparallel implants are commonly encountered in clinical situations. For this reason, the investigators evaluated the nonparallel condition, with some finding no significant difference between the accuracy of the closed tray technique and unsplinted open tray technique at up- to -15 degrees of angulation was seen [32-33].

Humphries et al. reported that the closed tray technique yielded a higher correlation to coordinate values on the definitive cast than open tray technique [19].

As cited previously one of the indication of close tray technique is angulated implants [6-7]. In this study angulation between inclined implants was 30 degrees, so these angulations may cause greater stress to the impression material once the tray is removed and generated lesser dimensional accuracy in casts in unsplinted open tray technique in comparison to close tray technique.

Comparing the results of experimental and semi-experimental studies, researchers have chosen various methods and set-ups. The difference in designing experimental models, measurement apparatus for distances under evaluation towards the reference points, and impression methods can make the comparison between these results rather difficult. Thus, it seems necessary to implement in vivo studies on this field to provide increased clinical generalization and reach the most accurate and the simplest impression methods for dental implants.

## Conclusion

With considering the limitations of this research, closedtray impression method on angulated implants seems to have a significant effect in reducing the dimensional changes in comparison to open tray method. Therefore, closed tray method is recommended due to its more simple application and lower impression time.

## References

[1] Seyedan K, Sazegara H, Kalalipour M, Alavi K (2008). Dimensional Accuracy of Polyether and Poly Vinyl Siloxane Materials for Different Implant Impression Technique. Res J Appl Scien.

[2] Rismanchian M, Monirifard R (2008). Implant impression, main patterns and modification: review. J Islam Socie Dent.

[3] Holst S, Blatz MB, Bergler M, Goellner M, Wichmann M (2007). Influence of impression material and time on the 3-dimensional accuracy of implant impressions. Quintessence Int.

[4] Choi JH, Lim YJ, Yim SH, Kim CW (2007). Evaluation of the accuracy of implant-level impression techniques for internal-connection implant prostheses in parallel and divergent models. Int J Oral Maxillofac Implants.

[5] Sazgara H, Nahidi R (2009). Comparison of implants’ impression techniques accuracy in close tray method using special and stock trays. Dent J Shahid Beheshti Univ Med Scien.

[6] Herbst D, Nel JC, Driessen CH, Becker PJ (2000). Evaluation of impression accuracy for osseointegrated implant supported superstructures. J Prosthet Dent.

[7] Vigolo P, Majzoub Z, Cordioli G (2003). Evaluation of the accuracy of three techniques used for multiple implant abutment impressions. J Prosthet Dent.

[8] Carr AB (1991). Comparison of impression techniques for a five-implant mandibular model. Int J Oral Maxillofac Implants.

[9] Brånemark PI (1983). Osseointegration and its experimental background. J Prosthet Dent.

[10] Brånemark PI, Zarb GA, Albrektsson T (1990). Tissue interated prostheses.

[11] Choi JH, Lim YJ, Yim SH, Kim CW (2007). Evaluation of the Accuracy of Implant- Level Impression Techniques for Internal Connection Implant Prostheses in Parallel and Divergent Models. Int Oral Maxillofac Implants.

[12] Walker MP, Ries D, Borello B (2008). Implant cast accuracy as a function of impression techniques and impression material viscosity. Int J Oral Maxillofac Implants.

[13] Del'Acqua MA, Arioli-Filho JN, Compagnoni MA, Mollo Fde A Jr (2008). Accuracy of impression and pouring techniques for an implant-supported prosthesis. Int J Oral Maxillofac Implants.

[14] Prithviraj DR, Pujari ML, Garg P, Shruthi DP (2011). Accuracy of the implant impression obtained from different impression materials and techniques: review. J Clin Exp Dent.

[15] Cabral LM, Guedes CG (2007). Comparative analysis of 4 impression techniques for implants. Implant Dent.

[16] Conrad HJ, Pesun IJ, DeLong R, Hodges JS (2007). Accuracy of two impression techniques with angulated implants. J Prosthet Dent.

[17] Daoudi MF, Setchell DJ, Searson LJ (2001). A laboratory inves-tigation of the accuracy of two impression techniques for single-tooth implants. IntJProsthodont.

[18] Walker MP, Ries D, Borello B (2008). Implant cast accuracy as a function of impression techniques and impression material viscosity. Int J Oral Maxillofac Implants.

[19] Humphries RM, Yaman P, Bloem TJ (1990). The accuracy of implant master casts constructed from transfer impressions. Int J Oral Maxillofac Implants.

[20] Naconecy MM, Teixeira ER, Shinkai RS, Frasca LC, Ce-rvieri A (2004). Evaluation of the accuracy of 3 transfer techniques for implant-supported prostheses with multiple ab-utments. Int J Oral Maxillofac Implants.

[21] Assif D, Fenton A, Zarb G, Schmitt A (1992). Comparative accuracy of implant impression procedures. Int J Periodontics Restorative Dent.

[22] Assif D, Marshak B, Schmidt A (1996). Accuracy of implant impression techniques. Int J Oral Maxillofac Implants.

[23] Vigolo P, Majzoub Z, Cordioli G (2003). Evaluation of the accuracy of threetechniques used for multipleimplantabutmentimpressions. J Prosthet Dent.

[24] Vigolo P, Fonzi F, Majzoub Z, Cordioli G (2004). An evaluation of impression techniques for multiple internal connection implant prostheses. J Prosthet Dent.

[25] Assif D, Nissan J, Varsano I, Singer A (1999). Accuracy of implant impression splinted techniques: effect of splinting material. IntJ OralMaxillofacImplants.

[26] Fenton A, Assif D, Zarb G, Schmitt A (1991). The accuracy of implant impression procedures. J Dent Res.

[27] Herbst D, Nel JC, Driessen CH, Becker PJ (2000). Evaluation of impression accuracy for osseointegrated implant support-ed superstructures. J Prosthet Dent.

[28] Hsu CC, Millstein PL, Stein RS (1993). A comparative analysis of the accuracy of implant transfer techniques. J Prosthet Dent.

[29] De La Cruz JE, Funkenbusch PD, Ercoli C, Moss ME, Graser GN, Tallents RH (2002). Verification jig for implant-supported prostheses: A comparison of standard impressions with verification jigs made of different materials. J Prosthet Dent.

[30] Burawi G, Houston F, Byrne D, Claffey N (1997). A comparison of the dimensional accuracy of the splinted and unsplin-ted impression techniques for the Bone-Lock implant sys-tem. J Prosthet Dent.

[31] Inturregui JA, Aquilino SA, Ryther JS, Lund PS (1993). Evalua-tion of three impression techniques for osseointegrated oral implants. J Prosthet Dent.

[32] Conrad HJ, Pesun IJ, DeLong R, Hodges JS (2007). Accuracy of two impression techniques with angulated implants. J Prosthet Dent.

[33] Carr AB (1992). Comparison of impression techniques for a two-implant 15-degree divergent model. Int J Oral Maxillofac Implants.

